# The Predictive Power of Religious Coping on Care Burden, Depression, Stress, and Anxiety of Parents of Pediatric Oncology Patients in Turkey

**DOI:** 10.1007/s10943-024-02096-3

**Published:** 2024-08-11

**Authors:** Remziye Semerci, Gülzade Uysal, Ayfer Açikgöz, Pınar Demirer

**Affiliations:** 1 School of Nursing, Koç University, Istanbul, Turkey; 2 Faculty of Health Sciences, Sakarya University of Applied Sciences, Sakarya, Turkey; 3 Faculty of Health Sciences, Osmangazi Eskisehir University, Eskişehir, Turkey; 4 Division of Pediatric Hematology and Oncology, Istanbul University, Istanbul, Turkey

**Keywords:** Pediatric oncology, Caregiver, Burden, Religious coping, Depression, Anxiety, Stress

## Abstract

This study aimed to determine the predictive power of religious coping of parents of children with cancer on caregiver burden, depression, anxiety, and stress in Turkey. It was designed as a descriptive and cross-sectional study, utilizing correlational analysis and regression models to explore associations between variables. Data were collected from 164 parents in the pediatric hematology-oncology clinics of a university hospital between November 2023 and March 2024. There was a negative correlation between caregiver burden score and negative and positive religious coping scores. Caregiver burden scores were positively correlated with depression, anxiety, and stress scores. Results indicated that caregiver burden, education level, employment status, family structure, family income, and age at diagnosis significantly predicted positive religious coping. For negative religious coping, caregiver burden, education level, family structure, and family income were significant predictors. This suggests that religious coping may help reduce caregiver burden, underscoring the importance of promoting constructive coping strategies to support caregivers' well-being.

## Introduction

Childhood cancers are a global health problem affecting children and their families worldwide (American Childhood Cancer Organization, 2022). When considering the impact of childhood cancer, it is essential to recognize the devastating nature of a cancer diagnosis, regardless of the treatment and resources available. This is particularly evident in resource-limited countries, where the relevant statistics are concerning. In addition, inequalities in children's access to health services exacerbate these concerns, which are prevalent in high-income and low-income countries (Johnston et al., 2021; Kebudi & Cakir, 2016; WHO, 2021a). According to the World Health Organization (WHO), the global burden of childhood cancer is indeed substantial, with approximately 400,000 children (aged 0–19 years) being diagnosed with cancer each year. Furthermore, the WHO estimates that there will be an expected 13.7 million new childhood cancer cases worldwide between 2020 and 2050 (WHO, 2021b; Atun et al., 2020).

Advances in childhood cancer treatment have significantly increased five-year survival rates, reaching 85% in developed countries (Kebudi & Alkaya, 2021). This rate indicates a significant improvement compared to the survival rate of 58% reported in the 1970s. The five-year survival rate for children and adolescents diagnosed with cancer in Turkey is 70.8% (Kutluk & Yeşilipek, 2019). However, despite these advances, cancer remains the second leading cause of death after accidents among children aged 1–14 years (American Cancer Society, 2021). In Turkey, between 4,000 and 4,500 new cases of pediatric cancer (in children aged 0–14 years) are diagnosed each year (Gheorghe et al., 2020). Unfortunately, these rates are predicted to continue to rise in Turkey and globally in the future.

## Care Burden, Depression, Stress, and Anxiety

Current treatments for childhood cancers have significantly increased survival rates, but these treatments often lead to negative physical, cognitive, and psychological side effects that affect the quality of life of both the child and their family. Although the diagnosis of cancer seems to impact children more, it also has a devastating negative impact on their families (Kong & Guan, 2019). The diagnosis comes as a shock to the parents, as they cannot believe that their child is facing a life-threatening illness such as cancer. In a state of shock, parents are burdened with many responsibilities related to their child's treatment, and these tasks place a significant burden of care on the family (Vitorino et al., 2018). In addition to the care burden, parents often have to deal with psychological problems such as guilt, frustration, sadness, grief, despair, hopelessness, anxiety, depression, and loneliness (Chong et al., 2019; Mowla et al., 2020). Consequently, there is an urgent need for strategies to improve caregivers' well-being. Given the burden of care and psychological challenges faced by caregivers as the prevalence of childhood cancer cases increases, it is crucial to take measures to support and improve caregivers' mental health (Kong & Guan, 2019).

## Religious Coping

The literature indicates that there is a relationship between religious or spiritual practices and human well-being (Mowla et al., 2020). Religious and spiritual practices are reported to play an important role in supporting cancer patients to manage psychological distress (Malhotra & Thapa, 2015; Mosher et al., 2015). This positive impact may extend to caregivers of pediatric oncology patients. Several studies show that religious coping practices are crucial in supporting parents to manage the struggles associated with caring for their children diagnosed with cancer (Abu-Raiya & Sulleiman, 2021; Khanjari et al., 2018; Mowla et al., 2020). In a study with parents of children with cancer in Iran, religious coping practices were found to help reduce the burden on the caregiver and reduce psychological distress (Hassankhani et al., 2019). Similarly, another study found that religious coping practices increased parents' ability to adapt to their children's cancer treatment and reduced their stress levels (Vitorino et al., 2018).

Considering this information, caring for a child diagnosed with cancer poses significant challenges for parents. The literature highlights that religious coping practices can reduce parents' caregiving burden. In this regard, this study aimed to determine the predictive power of religious coping in terms of caregiving burden, depression, stress, and anxiety among parents of pediatric oncology patients in Turkey.

## Research Questions

1. Is there a relationship between parents' depression, stress, and anxiety levels and their religious coping?
2. Is there a relationship between parents' care burden levels and religious coping?
3. What is the predictive power of parents' religious coping on their levels of depression, stress, anxiety, and care burden?

## Methods

### Study Type

This study aimed to determine the predictive power of religious coping in terms of caregiving burden, depression, stress, and anxiety among parents of pediatric oncology patients in Turkey. This study was conducted as a descriptive, correlational, and cross-sectional study.

### Study Design and Setting

This study was conducted between November 2023 and March 2024. Data were collected from parents of children aged 0–18 years who received care in a university hospital's pediatric hematology-oncology clinics and outpatient departments.

### Study Participants

The study sample consisted of parents of pediatric oncology patients. The sample size was calculated using the G*Power program (Faul et al., 2007). Due to the lack of similar studies, the relationship between parents' responses to the 'Caregiver Burden Scale,' 'Depression Anxiety Stress Scale', and 'Religious Coping Scale' was expected to be weak to moderate. Accordingly, it was aimed to reach 164 parents with an effect size of |ρ| = 0.20, a two-tailed α error probability of 0.05, and a power of 0.80 (1-β error probability) with the point two-series correlation model.

*Inclusion criteria* having a child with a diagnosis of pediatric oncology, having the child be diagnosed at least one month ago, continuing active treatment, speaking Turkish, and volunteering to participate in the study.

*Exclusion criteria* having a child in the terminal period, submitting incomplete data, having auditory or visual problems, receiving psychological treatment, and wanting to withdraw from the study at any stage of the study.

### Ethical Approach

Ethics committee approval (Approval Number: 2023/1872) and institutional permission were obtained for the conduct of the study. The researchers explained the purpose and scope of the study to the parents. It was ensured that the participants' confidentiality and participation were entirely voluntary. Face-to-face interviews were conducted with the parents during the data collection process, and written informed consent was obtained. Parents were informed that they could withdraw from the study without any justification. All study procedures were carried out following the principles stated in the Declaration of Helsinki.

### Data Collection Tools

Data were collected through ‘The Descriptive Information Form,’ ‘Caregiver Burden Scale,’ ‘Depression Anxiety Stress Scale,’ and ‘Religious Coping Scale’.

*Descriptive information form* was developed by the researchers consists of a total of 18 questions, including 17 questions about the socio-demographic characteristics of children and parents (age, gender, family structure, employment status, education level, income status, number of children, presence of other children with chronic diseases, etc.) and one question about parents' religious coping methods.

*The caregiver burden scale* was developed by Zarit et al., (1980). The scale was developed to assess the careburden of the caregivers. A validity and reliability study was conducted in Turkey by İnci and Erdem (2006). The scale consists of 22 items that mainly focus on social and emotional burden. The scale consists of a 5-point Likert-type scale ranging from 0 to 4, including ‘never’, ‘rarely’, ‘sometimes’, ‘often’ and ‘always’. The Cronbach's alpha value of the scale was reported as 0.95. Item-total correlation coefficients ranged from moderate to very strong (0.43–0.85), and the test–retest reliability coefficient was 0.90. A minimum score of 0 and a maximum score of 88 can be obtained from the scale. The categorization of the scores: 0–20 indicates little or no burden, 21–40 indicates mild to moderate burden, 41–60 indicates moderate to severe burden, and 61–88 indicates extreme burden. Higher scores indicate more caregiving burden. In this study, Cronbach's alpha value was found to be 0.88.

*Depression, anxiety, and stress scale* was developed by Lovibond and Lovibond (1995) and consists of 42 items. The scale is a 4-point Likert-type instrument in the form of ‘0 = not at all suitable for me’, ‘1 = somewhat suitable for me’, ‘2 = usually suitable for me’ and ‘3 = completely suitable for me’. The scale has a total of 42 items, 14 of which belong to depression, 14 to anxiety and 14 to stress dimensions (Tümkaya et al., 2009).

The 21-item DASS scale used in the study was taken from the studies of Henry and Crawford (2005) and Mahmoud et al (2012). In this scale (DASS-21), there are 21 questions, 7 questions each to measure depression, stress and anxiety dimensions. The Turkish validity and reliability study of the short form of the scale was conducted by Yılmaz et al. (2017) Cronbach's alpha value was found to be 0.85 for depression, 0.88 for anxiety, and 0.85 for stress.

*The religious coping scale* was developed by Abu Raiya et al., (2008). The Turkish validity and reliability were performed by Ekşi and Sayın (2016). The scale's Cronbach alpha was 0.91 for the positive religious coping sub-dimensions and 0.86 for the negative religious coping sub-dimensions. The scale consists of two subscales and 10 items. The subscales are "Positive Religious Coping" and "Negative Religious Coping". Positive religious coping includes items 1, 2, 3, 4, 5, 6, and 7, and negative religious coping includes items 8, 9, and 10.

There are no reverse-coded items in the scale. Positive and negative religious coping scores are calculated separately (Ekşi & Sayın, 2016). A total religious coping score is not obtained from the scale. The scale's Cronbach alpha was 0.91 for the positive religious coping subscale and 0.86 for the negative religious coping subscale. In this study, the Cronbach alpha of positive religious coping sub-dimensions was 0.89; for negative religious coping sub-dimensions was 0.90.

### Data Collection Procedure

Study data were collected in pediatric haematology-oncology and outpatient clinics of a university hospital between November 2023 and March 2024. Parents were included in the study according to the eligibility criteria, and those who were eligible were informed about the study. The study's purpose and the participants' responsibilities were explained to the parents through an informed consent form. Data collection took approximately 10–15 min for the parents to complete the questionnaires and scales.

### Data Analysis

Data were analyzed using the licensed IBM SPSS 28 package program (IBM SPSS Statistics for Windows, Version 28.0. Armonk, NY: IBM Corp.). Descriptive characteristics were evaluated using numbers, percentages, mean, and standard deviation. The homogeneity of the distribution was assessed with the Kolmogorov–Smirnov test. The Spearman correlation test was employed to examine the relationships between descriptive characteristics and the mean of the scale scores. The predictive power of parents' scores on the ‘Caregiver Burden Scale,’ ‘Depression Anxiety Stress Scale,’ and ‘Religious Coping Scale’ for risk scores was assessed using logistic regression. The logistic regression analysis included independent variables with a VIF value of < 10, a tolerance value of < 0.2, and a state index value of < 15. Results were analyzed at a 95% confidence interval, and significance was determined at *p* < 0.05.

## Results

The descriptive characteristics of the parents are presented in Table 1. The mean age of the parents was 36.38 ± 7.56 years, 50.6% of the parents were fathers, 38.4% of them had primary school education, 53% of them did not work, 77.4% of them had a nucleus family structure, 53.4% of had income less than expenditure, 15.2% of them had chronic diseases, and 4.9% of them had another child with a chronic illness (Table 1).

**Table 1:** The descriptive characteristics of the parents (n = 164)

Variables	M ± SD	
Parent’s age	36.38 ± 7.56	
Proximity to child	n	%
Mother	81	49.4
Father	83	50.6
*Education*		
Illiterate	14	8.5
Primary school	63	38.4
High school	57	34.8
University	30	18.3
*Employment status*		
Working	77	47.0
Not working	87	53.0
*Family structure*		
Nucleus	127	77.4
Extended	37	22.6
*Family income*		
Income less than expenditure	87	53.4
Income equals expenditure	64	39.3
Income more than expenditure	13	7.4
*Having chronic diseases*		
Yes	25	15.2
No	139	84.8
*Having another child with a chronic illness*		
Yes	10	4.9
No	154	95.1

M, mean; SD, standard deviation

The descriptive characteristics of the pediatric oncology patients are presented in Table 2. The mean age of the children was 7.92 ± 8.96 years, and the diagnosis duration was 6.28 ± 4.60 months. More of the children were male (64%), 34.8% of them diagnosed with leukemia. Most of the children received chemotherapy (97%), 26.4% received radiotherapy, and 32.9% received surgery (Table 2).

**Table 2:** The descriptive characteristics of the pediatric oncology patients (n = 164)

Variables	M ± SD	
Children’s age	7.92 ± 8.96	
Age at diagnosis (months)	6.28 ± 4.60	
	n	%
*Gender*		
Female	59	36.0
Male	105	64.0
*Diagnosis*		
Leukemia	57	34.8
Lymphoma	29	17.7
Cranial tumor	28	17.1
Osteosarcoma	8	4.9
Neuroblastoma	12	7.3
Soft tissue sarcomas	19	11.6
Wilms tumor	11	6.7
*Receiving chemotherapy*		
Yes	159	97.0
No	5	3.0
*Receiving radiotherapy*		
Yes	43	26.4
No	121	73.6
*Receiving surgery*		
Yes	54	32.9
No	110	67.1
*Child's school attendance status*		
Attending school	35	26.5
School suspended	82	62.1
Dropped out of school	15	11.4
*The child has a different disease*		
Yes	140	86.4
No	23	13.6

M, mean; SD, standard deviation

**Fig. 1:**
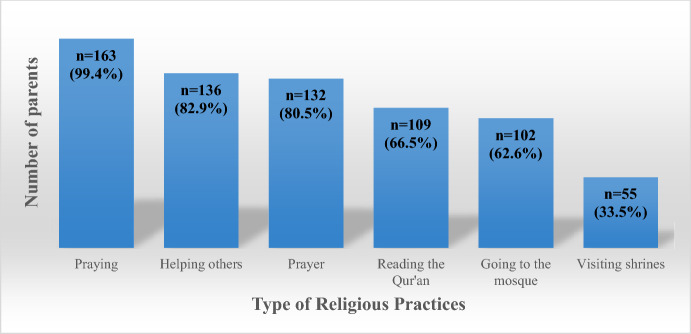
Religious practices of parents (n = 164)

Figure 1 illustrates the religious practices of parents participating in the study. It shows that while an even higher percentage, 99.4% (163 out of 164), perform praying rituals, 98.8% of the parents (162 out of 164) believe in a religion, indicating a high level of religious affiliation among participants. Additionally, a significant portion of parents, 82.9% (136 out of 164), participate in helping others, and 80.5% of the parents (132 out of 164) engage in regular prayer. Reading the Qur'an is practiced by 66.5% of the parents (109 out of 164), and 62.6% (102 out of 164) attend the mosque. Furthermore, 33.5% (55 out of 164) visit places of worship, such as shrines. These findings indicate a strong engagement in various religious and spiritual activities among the parents in the study (Fig. 1).

The correlation between Religious Coping Scale, Caregiver Burden Scale, and Depression, Anxiety, and Stress Scale was presented in Table 3. There was a positive correlation between Positive Religious Coping and Negative Religious Coping (r = 0.279). There was a negative correlation between the Caregiver Burden Scale and Positive Religious Coping (r = − 0.157) and between the Caregiver Burden Scale score and Negative Religious Coping (r = − 0.167). There was a positive correlation between the Depression score and the Caregiver Burden Scale score (r = 0.467). There was a positive correlation between Anxiety and the Caregiver Burden Scale score (r = 0.432). There was a positive correlation between Anxiety and the Depression score (r = 0.666). There was a positive correlation between Stress and Caregiver Burden Scale score (r = 0.491). There was a positive correlation between Stress and Depression (r = 0.681) and between Stress and Anxiety scores (r = 0.734) (Table 3).

**Table 3:** Correlation between scale scores (n = 164)

Variables		1	2	3	4	5	6
Religious coping scale	1. Positive religious coping	1.000					
	2. Negative religious coping	0.279*	1.000				
	3. Caregiver burden scale	− 0.157*	− 0.167*	1.000			
Depression anxiety stress scale	4. Depression	− 0.147	0.089	0.467*	1.000		
	5. Anxiety	0.143	0.006	0.432*	0.666*	1.000	
	6 Stress	− 0.110	− 0.031	0.491*	0.681*	0.734*	1.000

^*^p < 0.05

Based on the relationship between variables in multiple regression analysis, the impact of sub-dimensions of the Religious Coping Scale scores for parents of children with cancer on various outcomes was assessed. For positive religious coping, the analysis revealed several important findings. The caregiver burden scale had a negative but not statistically significant effect on positive religious coping (B = − 0.044, *p* = 0.064). Similarly, the depression (B = − 0.131, *p* = 0.061), anxiety (B = − 0.126, *p* = 0.068), and stress (B = − 0.099, *p* = 0.159) sub-dimensions of DASS-21 did not significantly predict positive religious coping. However, proximity to the child (mother) was a significant predictor, with a negative relationship (B = − 1.807, *p* = 0.004). Education level (illiterate) also showed a significant negative effect on positive religious coping (B = -1.477, *p* < 0.001), while employment status (not working) (B = 1.803, *p* = 0.004), family structure (extended) (B = 1.770, *p* = 0.013), and family income (income less than expenditure) (B = − 1.529, *p* = 0.015) were significant predictors. Age at diagnosis also significantly predicted positive religious coping with a negative relationship (B = − 0.165, *p* = 0.002) (Table 4).

**Table 4:** The religious coping score of the parents predicted their depression, anxiety, stress, and caregiver burden scores (n = 164)

Variable		Positive religious coping					
		B	SE	*β*	t	*p*	
Caregiver burden scale		− 0.044	0.022	− 0.22	− 2.026	0.064	R = 0.157; R^2^ = 0.025; F = 3.104; p = 0.064; DW = 1.933
DASS-21	Depression	− 0.131	0.069	− 0.147	− 1.889	0.061	R = 0.147; R^2^ = 0.022; F = 3.568; p = 0.061; DW = 1.990
	Anxiety	− 0.126	0.069	− 0.143	− 1.837	0.068	R = 0.143; R^2^ = 0.020; F = 3.376; p = 0.068; DW = 1.975
	Stress	− 0.099	0.070	− 0.110	− 1.415	0.159	R = 0.110; R^2^ = 0.012; F = 2.001; p = 0.159; DW = 1.931
Proximity to child (mother)		− 1.807	0.612	− 0.226	− 2.951	0.004	R = 0.226; R^2^ = 0.051; F = 8.711; p = 0.004; DW = 2.018
Education (illiterate)		− 1.477	0.339	− 0.324	− 4.360	< 0.001	R = 0.324; R^2^ = 0.105; F = 10.009; p < 0.001; DW = 1.872
Employment status (not working)		1.803	0.613	0.225	2.938	0.004	R = 0.225; R^2^ = 0.051; F = 8.635; p = 0.004; DW = 2.033
Family structure (extended)		1.770	0.707	0.193	2.504	0.013	R = 0.193; R^2^ = 0.037; F = 6.270; p = 0.013; DW = 1.889
Family income (income less than expanditure)		− 1.529	0.486	− 0.241	− 3.145	0.015	R = 0.214; R^2^ = 0.058; F = 9.892; p = 0.015; DW = 1.928
Age at diagnosis		− 0.165	0.067	− 0.189	− 2.456	0.002	R = 0.189; R^2^ = 0.036; F = 96.031; p = 0.002; DW = 1.943

		Negative religious coping					
		B	SE	*β*	t	*p*	
Caregiver burden scale		− 0.040	0.019	− 0.167	− 2.157	0.032	R = 0.167; R^2^ = 0.028; F = 3.369; p = 0.032; DW = 1.796
DASS-21	Depression	0.067	0.059	0.089	1.131	0.260	R = 0.089; R^2^ = 0.008; F = 0.007; p = 0.260; DW = 1.782
	Anxiety	0.005	0.059	0.006	0.082	0.935	R = 0.006; R^2^ = 0.000; F = 8.711; p = 0.935; DW = 1.771
	Stress	− 0.023	0.060	− 0.031	− 0.393	0.695	R = 0.031; R^2^ = 0.001; F = 0.154; p = 0.695; DW = 1.772
Education (illiterate)		− 1.241	0.288	− 0.321	− 4.309	< 0.001	R = 0.321; R^2^ = 0.103; F = 18.568; p < 0.001; DW = 1.716
Family structure (extended)		2.170	0.588	0.279	3.692	< 0.001	R = 0.279; R^2^ = 0.078; F = 13.629; p < 0.001; DW = 1.699
Family income (income less than expanditure)		− 1.098	0.416	− 0.204	− 2.642	0.009	R = 0.204; R^2^ = 0.042; F = 6.982; p = 0.009; DW = 1.676

B, unstandardized beta; SE, standard error; β, standardized beta β; R, correlation; R^2^, correlation coefficient (explained variance ratio), F, model statistics; p, level of significance; FINPED II, family inventory of needs pediatric II; DASS-21, depression, anxiety and stress scale; DW, durbin Watson

For negative religious coping, the caregiver burden scale was a significant predictor with a negative relationship (B = − 0.040, *p* = 0.032). Depression (B = 0.067, *p* = 0.260), anxiety (B = 0.005, *p* = 0.935), and stress (B = − 0.023, *p* = 0.695) sub-dimensions of DASS-21 were not significant predictors of negative religious coping. Education level (illiterate) was a significant negative predictor (B = − 1.241, *p* < 0.001), while family structure (extended) (B = 2.170, *p* < 0.001) and family income (income less than expenditure) (B = − 1.098, *p* = 0.009) were also significant predictors of negative religious coping. These findings highlight the complex interplay between various factors and religious coping strategies among parents of children with cancer (Table 4).

## Discussion

This study aims to determine the predictive power of religious coping in terms of caregiving burden, depression, stress, and anxiety among Turkish parents of pediatric oncology patients. The study determined that most parents (98.8%) believed in a religion, 80.5% prayed, 99.4% prayed, and 66.5% read the Qur'an. 82.9% of the respondents stated that they helped others, and 33.5% visited places of worship (Fig. 1). People struggle with problems such as death, divorce, and illness throughout their lives and try to cope with them. Thus, they aim to regain control of life and gain a sense of meaning in life. One of the most essential strategies people use to cope with these stressful situations is religion. Religion helps people to overcome problems by giving them courage and meaning. It helps to maintain psychological well-being by providing patience and acceptance in a situation that cannot be coped with (Abanoz, 2020).

In a study, it was determined that 80% of relatives of cancer patients had positive contributions from using religious coping strategies. It was determined that prayer and patience were the most used religious coping strategies, followed by fate, gratitude, and surrendering to trial (Akbulut-Şahin, 2019). The high number of those who believe in a religion, pray, and prayer among the participants in the current study may be related to the characteristics of the population in which the study was conducted. People's most valuable assets in life are their children. The fact that their children are struggling with a complex disease such as cancer may have led parents to fulfill religious responsibilities more. Another reason for this result may be that religious people are more willing to participate in this research.

The study's results indicate a positive relationship between Positive Religious Coping and Negative Religious Coping scores, with both types of coping increasing or decreasing in parallel, suggesting an intriguing dynamic in the context of religious coping strategies. This phenomenon may be attributed to the specific characteristics of the scale utilized in the study, particularly the absence of items related to distancing from religion within the negative religious coping items (Ekşi and Sayın (2016). Instead, the negative religious coping items focused on attributing problems to sins committed by individuals and the potential for punishment by God for perceived shortcomings in faithfulness (Ano & Vasconcelles, 2005). In Islamic beliefs, the world is viewed as a testing ground where individuals face trials and tribulations as a means of assessing their patience, prompting reflection on their actions, and potentially serving as a form of retribution for sins committed in this life rather than the afterlife, as outlined in the Qur'an (Nikfarid et al., 2018; Saari et al., 2022). Therefore, individuals who adhere to Islamic beliefs may resonate with positive and negative religious coping strategies, as both are considered valid within the framework of their faith (Mazhari et al., 2021).

The positive relationship observed between Positive Religious Coping and Negative Religious Coping in the study may reflect the multifaceted nature of religious coping within an Islamic context. While Positive Religious Coping may involve seeking solace, guidance, and strength from religious beliefs and practices, Negative Religious Coping could encompass guilt, fear of divine retribution, or a sense of accountability for one's actions based on religious teachings (Dolcos et al., 2021). The co-occurrence of these coping strategies suggests a complex interplay between seeking comfort and confronting challenges within the religious worldview of Islam (Ano & Vasconcelles, 2005). Future research could delve deeper into the nuances of religious coping among individuals of different faith traditions, exploring how specific religious beliefs and doctrines shape coping mechanisms in response to life stressors. By examining the interplay between positive and negative religious coping strategies within diverse religious contexts, researchers can better understand how individuals draw upon their faith to navigate adversity and find meaning in challenging circumstances.

This study found a negative correlation between the Caregiver Burden Scale and Positive and Negative Religious Coping (Table 3). Positive religious coping is associated with lower levels of psychological distress. In comparison, negative religious coping is reported to be associated with higher levels of distress among parents of children with cancer (Dolan et al., 2021). This suggests that the way parents use religious coping mechanisms may significantly affect their psychological well-being when caring for a child with cancer. Research has shown that caregivers who use coping approaches emphasizing problem-focused techniques tend to reduce caregiver burden and better adapt to the situation (Deribe et al., 2023; Dolan et al., 2021; Liu et al., 2007). Caregivers who employ problem-focused coping techniques, which involve actively addressing and managing stressors, tend to experience lower burden levels and demonstrate better adaptation to their caregiving roles. The findings from these studies underscore the significance of religious coping mechanisms and coping strategies in influencing the psychological well-being and caregiver burden experienced by parents of children with cancer. Healthcare professionals working with this population should consider the role of religious coping and encourage the use of positive coping strategies to support caregivers in managing their stress and promoting better psychological outcomes.

The positive correlation observed between the Depression, Anxiety, Stress score and the Caregiver Burden Scale score among parents of children with cancer suggests that there is a significant relationship between parents' psychological well-being and the burden they experience while caring for their sick children (Table 4). This finding is consistent with recent literature investigating the impact of parental mental health on caregiving in the context of childhood cancer. Studies such as Salem et al. (2019) have highlighted high levels of psychological distress, anxiety, and depression in parents of children with cancer, particularly in the early years following diagnosis. This suggests that parents' emotional well-being is intricately linked to their challenges when caring for their sick children. Link and Fortier (2016) demonstrated that parental anxiety significantly predicted various aspects of the child's quality of life, suggesting that parental mental health and child well-being are interconnected. Luo et al. (2022) discussed the role of resilience in parents of children with cancer. They showed that resilience can positively affect parents' mental health and is negatively associated with depressive symptoms and post-traumatic stress disorder.

This underlines the importance of resilience in mitigating the adverse effects of caregiving stress on parental mental health. Rismawati and Paramastri (2020) investigated the role of religious coping as a moderator of the relationship between psychological burden and quality of life among caregivers of individuals with cancer. Although the study did not find that religious coping significantly moderated this relationship, it highlights the complex interaction between coping mechanisms, psychological burden, and quality of life in caregivers. Norberg et al. (2008) found that anxiety, depression, and post-traumatic stress symptoms of parents in childhood cancers were higher than healthy parents. Wang et al. (2017) concluded that the burden of care was high in parents of children with newly diagnosed acute lymphoblastic leukemia and that parents' anxiety decreased as the burden of care decreased. All these results are important in terms of showing how valuable each intervention to reduce the burden of caregiving in families with sick or disabled children is. In this way, the rates of anxiety, stress and depression in parents can be reduced. In conclusion, the positive correlation between indicators of parental mental health and caregiver burden in the context of childhood cancer underlines the need for comprehensive support systems that address the psychological well-being of parents caring for sick children. The literature suggests that interventions focussing on resilience, coping strategies, and mental health support can play an essential role in improving the overall well-being of parents facing the challenges of caring for children with cancer.

The results of the study indicate that parents' depression, anxiety, stress, and caregiver burden scores did not significantly affect the ‘Positive Religious Coping’ score. However, an increase in caregiver burden scores significantly predicted the ‘Negative Religious Coping’ score, accounting for 2.8% of its variation. These findings align with previous research showing an association between caregiver burden and negative religious coping (Cheang, 2023). Negative religious coping is linked to anxiety and depression, whereas positive religious coping has a lesser impact on mental health (Francis et al., 2019). Studies have also reported that negative coping practices are related to anxiety disorders and an increased risk of major depression  (Asano et al., 2021).  Conversely, religious coping has been associated with reduced anxiety and depression scores (Dolcos et al., 2021).

The study further highlights those factors such as proximity to the child, education level, employment status, family structure, and family income significantly influence religious coping strategies. These findings underscore the importance of considering these variables when developing interventions to support caregivers. The severity and duration of the stressor also play crucial roles in the effectiveness of coping strategies (Maier et al., 2022). The study aligns with previous findings that religiosity during the stress of illness can be associated with either positive or negative religious coping among caregivers (Asano et al., 2021; Dolcos et al., 2021; Maier et al., 2022). Promoting positive coping mechanisms is essential for enhancing caregivers' well-being, as these strategies can mitigate the adverse effects of caregiver burden.

## Limitations

Despite the valuable insights this study provides, some limitations must be acknowledged. The cross-sectional design limits the ability to establish causality between religious coping and psychological outcomes. Longitudinal studies are needed to elucidate the temporal dynamics of these relationships. The study sample was drawn from a single cultural and geographical context, which may limit the generalisability of the findings to other settings or populations. Cultural factors play an essential role in religious coping strategies, and future research should consider a more diverse sample to increase the external validity of the findings.

## Conclusion

This study contributes to the understanding of the interaction between religious coping mechanisms and the psychological health of parents caring for children with cancer. Our findings reveal that while positive religious coping does not significantly affect levels of depression, anxiety, stress, and caregiver burden, an increase in caregiver burden is a predictor of negative religious coping. This suggests that religious coping strategies play a nuanced role in caregivers' psychological resilience or vulnerability, highlighting the complex nature of spirituality in pediatric oncology care. The association between caregiver burden and negative religious coping underscores the importance of supporting caregivers on their spiritual journey, recognizing that negative religious coping may indicate increased psychological distress.

The findings from this study underline the need for a holistic approach in clinical settings, particularly in paediatric oncology, where the spiritual well-being of caregivers significantly influences overall mental health. Clinicians should conduct routine assessments of both the emotional and spiritual health of caregivers and identify the nature of their religious coping mechanisms. There is a critical need to develop support programmes that promote positive religious coping strategies. These programmes could include spiritual counselling and support groups facilitated by chaplains or spiritual leaders, designed to promote constructive spiritual practices. In addition, healthcare providers should receive targeted training to better understand the role of spirituality in mental health, enabling them to effectively integrate this aspect into their therapeutic approach. Finally, there is a call for the design of specific interventions aimed at reducing negative religious coping and associated psychological distress, which may include directly addressing underlying spiritual crises and conflicts. By implementing these strategies, health professionals can better support caregivers, reduce their burden and increase their overall psychological resilience.
